# Distinct effects of general psychological distress and COVID-19-specific fear during pregnancy on gestational age and infant birth weight

**DOI:** 10.3389/fgwh.2026.1653126

**Published:** 2026-02-13

**Authors:** Li Chen, Cheng-Han Li, Jing-Jing Xie, Qian-Nan Ruan, Bing Chen, Dong-Mei Lin

**Affiliations:** 1 Wenzhou People’s Hospital, Wenzhou, China; 2 Third Affiliated Hospital of Wenzhou Medical University, Wenzhou, China; 3 Wenzhou Seventh People’s Hospital, Wenzhou, China

**Keywords:** birth weight, COVID-19, gestational age, perinatal mental health, psychological distress

## Abstract

**Objective:**

To disentangle the independent and shared contributions of maternal general psychological distress (GPD) and COVID-19-specific fear (CSF) during pregnancy to gestational age at birth and infant birth weight.

**Methods:**

This study utilized secondary data from the prospective Canadian “Pregnancy during the COVID-19 Pandemic” cohort. The final analytic sample comprised 5,658 pregnant individuals. GPD was a latent variable indicated by the Edinburgh Postnatal Depression Scale (EPDS) and Patient-Reported Outcomes Measurement Information System (PROMIS) Anxiety scores. CSF was a latent variable indicated by three items assessing pandemic-related fears for self and baby. Structural equation modeling (SEM) examined associations with gestational age and birth weight, controlling for maternal age, income, and education.

**Results:**

The SEM demonstrated good fit. Higher GPD independently predicted shorter gestational age (standardized *β* = –.048, *p* = .002) but not lower infant birth weight (*p* = .632) after accounting for CSF. Higher CSF independently predicted both shorter gestational age (standardized *β* = –.058, *p* < .001) and lower infant birth weight (standardized *β* = –.058, *p* < .001), controlling for GPD and covariates. GPD and CSF were positively correlated (standardized covariance = .419, *p* < .001).

**Conclusion:**

COVID-19-specific fear is a unique and significant risk factor for shorter gestational age and lower infant birth weight, distinct from general psychological distress. These findings highlight the need to assess and address pandemic-specific fears in perinatal populations to mitigate adverse birth outcomes during public health crises. Targeted interventions for specific fears may be necessary beyond general mental health support.

## 1 Introduction

The perinatal period, spanning pregnancy through the first postpartum year, represents a critical window of both heightened vulnerability and profound opportunity for shaping the long-term health trajectories of mothers and their infants (1). It is well-established that maternal psychological distress during pregnancy, encompassing conditions such as depression and anxiety, is a significant risk factor for adverse birth outcomes (2). These include, but are not limited to, preterm birth and low birth weight, both of which carry substantial implications for neonatal morbidity and long-term child development (3). The mechanisms linking prenatal distress to adverse birth outcomes are understood to be multifaceted, involving a complex interplay of biopsychosocial factors. These include potential dysregulation of the hypothalamic-pituitary-adrenal (HPA) axis leading to altered cortisol rhythms, heightened systemic inflammatory processes, and modifications in maternal health behaviors such as nutrition, sleep, and substance use (4). While the impact of such general prenatal distress is extensively documented, the emergence of unique, large-scale societal stressors introduces novel complexities to understanding and mitigating risks to perinatal health.

The COVID-19 pandemic, declared a global health emergency in 2020, presented an unprecedented and pervasive stressor, profoundly impacting daily life, healthcare systems, and psychological well-being worldwide (5). Pregnant individuals rapidly emerged as a population experiencing heightened vulnerability during this crisis. This vulnerability was multifaceted, stemming from heightened concerns about viral transmission to themselves and their fetuses, significant disruptions to the continuity and nature of prenatal and postnatal care (e.g., cancelled appointments, shifts to telemedicine, restricted partner presence), increased social isolation due to public health measures like lockdowns, and widespread economic anxieties linked to job insecurity or financial instability (6). Consequently, a substantial body of research conducted during the pandemic has consistently documented markedly elevated rates of anxiety and depression among pregnant individuals when compared to pre-pandemic cohorts, underscoring the pandemic's considerable toll on maternal mental health (7).

The profound psychological impact of the COVID-19 pandemic on pregnant individuals necessitates a nuanced understanding, distinguishing between an exacerbation of general psychological distress (i.e., broad symptoms of anxiety and depression) and the emergence of pandemic-specific fear. COVID-19-specific fear (CSF) pertains to direct concerns about the virus's impact on the pregnant individual, their unborn baby, and the healthcare experience during the pandemic—such as fear of nosocomial infection, fear of adverse birth outcomes due to COVID-19 infection, or worry about restricted support during childbirth (8). The theoretical rationale for this distinction is compelling. Firstly, the cognitive content differs significantly: general anxiety involves a wider array of worries often lacking a singular, immediate external threat, whereas CSF is thematically focused on the direct and indirect threats posed by the pandemic (9). Secondly, the underlying neurobiological and physiological mechanisms linking distress to health outcomes might diverge; acute, threat-specific fear is more likely to trigger heightened sympathetic nervous system activity and pronounced fight-or-flight responses, potentially distinct from the more chronic neuroendocrine (e.g., HPA axis) and inflammatory dysregulations often associated with generalized anxiety or depression (10). This aligns with established psychopathological distinctions between fear as an immediate response to a present or imminent threat, and anxiety as a future-oriented state of apprehension about potential diffuse threats. Thirdly, the implications for intervention strategies differ: addressing specific fears about viral exposure or healthcare uncertainties necessitates targeted psychoeducation, risk communication, and coping strategies distinct from broader psychotherapeutic or pharmacological treatments for clinical anxiety or depression (11).

Despite the intuitive and theoretical importance of distinguishing between general psychological distress and CSF, their independent and potentially interactive contributions to tangible birth outcomes have remained insufficiently explored. Much of the early pandemic research focused on overall distress levels, with fewer studies employing robust methodologies capable of modeling these as distinct yet related psychological constructs and examining their unique predictive utility for perinatal health indicators (12). Addressing this gap is crucial for a more precise understanding of risk pathways during large-scale health crises.

To address these complexities, the current study utilized secondary data from the large, prospective Canadian “Pregnancy during the COVID-19 Pandemic” national cohort study. Large prospective cohort studies, particularly those initiated during unique exposure periods like a pandemic, are invaluable for delineating temporal associations between early-life exposures and subsequent health outcomes, minimizing recall bias. The primary objective of this research was to disentangle the unique and shared contributions of maternal general psychological distress and CSF during pregnancy to key infant birth outcomes, namely gestational age at birth and infant birth weight, while statistically controlling for established demographic and obstetric covariates. We employed structural equation modeling (SEM), a sophisticated statistical approach well-suited for examining complex interrelationships among latent and observed variables, allowing for the simultaneous estimation of direct and indirect effects and the modeling of distinct but correlated constructs.

Based on the existing literature on prenatal stress and the unique context of the pandemic, we hypothesized that: (1) higher levels of general psychological distress during pregnancy would be independently associated with shorter gestational age at birth and lower infant birth weight, and (2) higher levels of CSF during pregnancy would also be independently associated with shorter gestational age at birth and lower infant birth weight, even after accounting for general psychological distress. Disentangling these specific effects is crucial for identifying distinct risk pathways and informing the development of targeted interventions and public health strategies to mitigate the adverse consequences of pandemic-related stress, and future large-scale stressors, on both maternal and infant health.

## 2 Method

### 2.1 Data source and participants

This study utilized secondary data from the Pregnancy during the COVID-19 Pandemic dataset, which is publicly available via the Open Science Framework (OSF; https://osf.io/ha5dp/). Detailed data collection procedures are described here (13). The PdP project is a large, ongoing prospective cohort study designed to examine the impact of the COVID-19 pandemic on pregnant individuals and their infants across Canada. Data were collected through online self-report questionnaires administered via the REDCap platform, available in both English and French. All participants provided informed consent, and the study received ethical approval from the University of Calgary Conjoint Health Research Ethics Board (CHREB; REB20-0500).

A total of 10,771 pregnant individuals were initially recruited. For the current analyses, we employed listwise deletion to handle missing data; that is, any participant with missing values in key demographic, psychological, or birth outcome variables was excluded from analyses. While this is a conservative approach that reduces sample size, it provides a transparent complete-case analysis, avoiding the additional assumptions required by methods such as multiple imputation. After applying this criterion, the final analytic sample consisted of 5,658 participants. We compared key demographic and outcome variables between participants with complete data (complete cases) and those with missing data (incomplete cases). The results indicate that women in the complete case group were slightly older than those in the incomplete case group (mean age 32.51 vs. 31.20 years, *p* < 0.001). Moreover, the proportion of English-speaking participants was higher in the complete case group (81.5% vs. 70.4%, *p* < 0.001), while the proportion of French-speaking participants was lower. Regarding delivery mode, the complete case group had a higher proportion of vaginal deliveries and a lower proportion of caesarean sections (*p* = 0.046). There was no significant difference between groups in terms of infant birth weight (3,413.29 g vs. 3,405.99 g, *p* = 0.705). These findings suggest the possibility of selection bias due to missing data, though the differences are not large.

### 2.2 Measures

**Demographic Variables include** maternal age, household income for 2019, and highest level of education attained. **Infant Outcomes** Included gestational age at birth, birth weight, delivery mode, neonatal intensive care unit (NICU) admission, and delivery date.

#### 2.2.1 Depression symptoms

Assessed using the Edinburgh Postnatal Depression Scale (EPDS), a widely validated instrument for measuring depression symptoms both prenatally and postpartum (14). The EPDS consists of 10 items scored on a 4-point scale (0–3), yielding total scores ranging from 0 to 30, with higher scores indicating more severe depressive symptoms. The Cronbach's alpha coefficient is close to 0.88 (15).

#### 2.2.2 Anxiety symptoms

Measured with the Patient-Reported Outcomes Measurement Information System (PROMIS) Anxiety Adult Short Form, which includes 7 items scored from 1 to 5, with T-score transformation in this data. Higher scores reflect greater anxiety symptoms. The PROMIS Anxiety scale has demonstrated high internal consistency in perinatal samples, with Cronbach's alpha coefficients of approximately 0.90 (16).

#### 2.2.3 Fear of COVID-19

Assessed using three slider items developed by the original “Pregnancy during the COVID-19 Pandemic” study team to capture pandemic-specific perinatal anxieties. These items were designed to have high face validity for the unique context of the pandemic. Each was scored from 0 (“not at all”) to 100 (“very much so”), with a midpoint anchor at 50 (“somewhat”). Questions assessed perceived threat to the participant's own life, perceived threat to the unborn baby's life, and worry about harm to the unborn baby due to COVID-19. In our model, these items served as indicators for the latent construct of COVID-19-Specific Fear.

### 2.3 Statistical analysis

Structural equation modeling (SEM) was conducted in R using the lavaan package (Rosseel, 2012) to evaluate the relationships between maternal general psychological distress (as indicated by EPDS and PROMIS Anxiety scores), COVID-19-specific fear (indicated by the three fear items), and infant birth outcomes, while controlling for maternal age, income, and education. The covariance between the two latent constructs was estimated. Model fit was assessed using standard indices, including CFI, TLI, RMSEA, and SRMR. All analyses used listwise deletion for missing data. The analysis code and further details are available in the supplementary files and the OSF project page.

## 3 Results

### 3.1 Sample characteristics

Descriptive statistics for key demographic, psychological, COVID-19-related, and infant outcome variables across major categorical groups are presented in Table 1. The final analytic sample included 5,658 participants. The majority of participants reported an annual household income between $40,000 and $124,999 and had at least some college education. Most births were vaginal deliveries, and the majority of infants did not require admission to the NICU. Mean scores for maternal age, depression (EPDS), anxiety (PROMIS), COVID-19-related fears, gestational age at birth, and birth weight are also provided by subgroup in Table 1.

**Table 1:** Descriptive statistics of key study variables by categorical groups in the PdP sample (*N* = 5,658).

Categorical variable	*n* (%)	Maternal age	EPDS	PROMIS anxiety	Threaten life	Threaten_Baby_Danger	Threaten_Baby_Harm	Delivery_GAbirth	Delivery_Birth_Weight
Household_Income_Prenatal									
<$20,000	260 (2.4%)	28.09 (6.27)	13.22 (5.91)	61.58 (8.46)	50.77 (29.64)	60.28 (28.86)	66.99 (30.27)	38.98 (1.8)	3,228.4 (623.99)
$20,000–$39,999	611 (5.7%)	29.27 (5.35)	12.82 (6.14)	60.75 (8.66)	47.73 (28.37)	58.31 (28.96)	66.72 (28.61)	39.13 (1.81)	3,372.04 (618.8)
$40,000–$69,999	1,433 (13.3%)	30.44 (4.82)	11.59 (5.79)	59.61 (8.32)	45.22 (26.49)	53.74 (27.15)	63.48 (28.23)	39.28 (1.67)	3,413.81 (568.28)
$70,000–$99,999	2,094 (19.4%)	31.2 (4.33)	10.53 (5.53)	58.56 (8.73)	42.39 (25.79)	52.03 (27.1)	63.63 (27.36)	39.38 (1.67)	3,431.49 (553.69)
$100,000-$124,999	1,956 (18.2%)	31.88 (3.88)	9.84 (5.18)	57.9 (8.03)	41.78 (24.63)	50.29 (26.09)	62.39 (26.85)	39.42 (1.51)	3,432.92 (523.36)
$125,000–$149,999	1,365 (12.7%)	32.61 (3.78)	9.71 (5.11)	57.56 (7.63)	40.48 (25.11)	48.9 (25.76)	62.03 (26.17)	39.35 (1.65)	3,412.86 (520.49)
$150,000–$174,999	1,139 (10.6%)	33.03 (3.34)	9.12 (5.06)	56.72 (8.23)	41.39 (24.91)	48.46 (25.68)	59.98 (26.72)	39.41 (1.55)	3,400.1 (533.21)
$175,000–$199,999	655 (6.1%)	33.41 (3.63)	8.92 (5.15)	56.77 (7.9)	40.06 (24.75)	46.92 (25.72)	60.52 (26.42)	39.45 (1.41)	3,404.24 (509.93)
≥$200,000	1,008 (9.4%)	34.18 (3.73)	8.88 (5.13)	56.7 (8.42)	39.46 (24.3)	46.89 (24.93)	60.13 (26.34)	39.21 (1.64)	3,392.49 (509.68)
Maternal_Education_Prenatal									
Less than high school diploma	142 (1.3%)	27 (6.03)	14.07 (6.48)	62.04 (8.61)	43.77 (30.56)	55.19 (31.19)	65.01 (31.28)	39.04 (1.45)	3,384.85 (559.37)
High school diploma	901 (8.4%)	29.08 (5.3)	11.86 (5.71)	60.1 (8.47)	44.52 (26.61)	55.51 (27.49)	64.54 (28.81)	39.18 (1.64)	3,442.33 (587.76)
College/Trade school	2,760 (25.6%)	30.98 (4.6)	11.15 (5.85)	58.78 (8.79)	44.09 (26.31)	53.87 (27.52)	64.11 (27.55)	39.21 (1.72)	3,397.38 (556.47)
Undergraduate degree	4,117 (38.2%)	32.07 (3.93)	9.88 (5.24)	57.89 (8.17)	42.42 (25.37)	50.27 (26.14)	62.58 (27.05)	39.38 (1.59)	3,420.73 (529.33)
Master's degree	1,889 (17.5%)	33.24 (3.6)	9.17 (5.06)	57.11 (7.8)	39.87 (24.63)	47.37 (25.47)	60.4 (26.38)	39.44 (1.54)	3,412.95 (515.67)
Doctoral degree	786 (7.3%)	34.19 (3.57)	8.83 (5.05)	56.96 (8.18)	39.57 (24.57)	46.35 (25.33)	58.73 (25.83)	39.36 (1.62)	3,385.55 (560.83)
Delivery_Mode									
vaginally	3,904 (36.2%)	32.21 (4.04)	9.65 (5.31)	57.56 (8.11)	40.82 (24.9)	49.07 (26.07)	61.33 (27.07)	39.48 (1.48)	3,421.44 (498.12)
Caesarian	1,633 (15.2%)	33.27 (4.29)	9.96 (5.29)	58.22 (8.22)	42.79 (25.3)	50.85 (26.08)	63.04 (27.03)	38.97 (1.94)	3,394.78 (621.81)
Delivery_NICU_stay									
No	4,986 (46.3%)	32.48 (4.13)	9.62 (5.25)	57.55 (8.13)	40.98 (24.89)	49.15 (26)	61.4 (27.19)	39.53 (1.26)	3,455.9 (479.47)
Yes	548 (5.1%)	32.92 (4.26)	10.83 (5.7)	59.58 (8.12)	45.23 (25.99)	53.64 (26.58)	65.72 (25.68)	37.49 (3.04)	3,029.74 (815.5)
Language									
English	8,154 (75.7%)	32.25 (4.3)	10.41 (5.4)	58.51 (8.25)	43.98 (25.1)	51.63 (26.23)	63.17 (27.35)	39.35(1.61)	3,424.29(535.34)
French	2,617 (24.3%)	30.55(4.54)	9.46(5.79)	56.82(8.59)	37.03(26.65)	48.3(27.84)	59.97(26.73)	39.31(1.61)	3,360.92(549.98)

**Figure 1:**
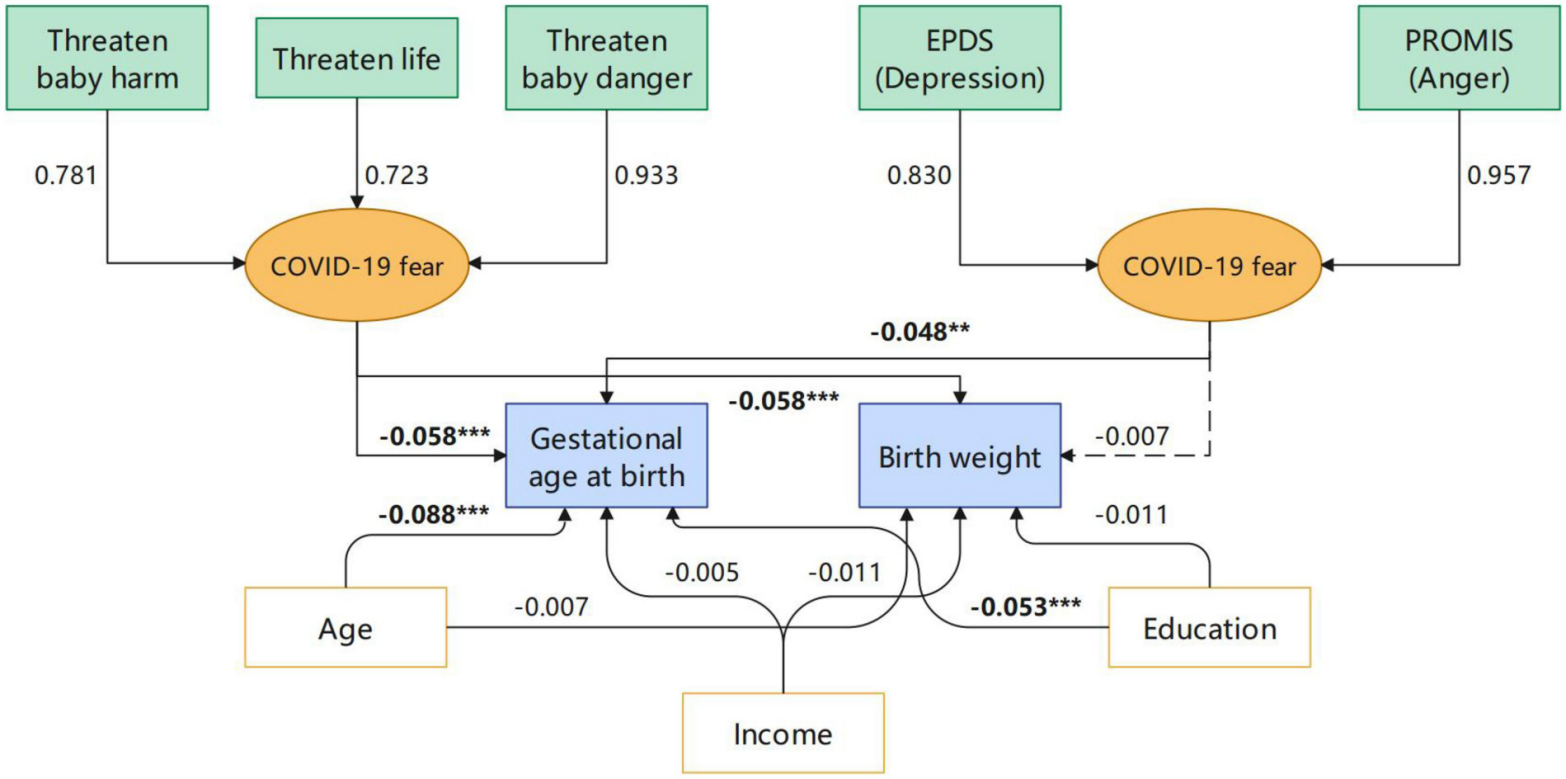
Structural equation model examining associations among general psychological distress, COVID-19-specific fear, and birth outcomes. Yellow nodes represent latent variables, green nodes represent observed variables, white nodes represent control variables, and blue nodes represent outcome variables. Arrow labels indicate standardized path coefficients. * denotes significance (*p < .05, **p < .01, ***p < .001).

The hypothesized SEM demonstrated good fit to the data (Figure 1). The chi-square test was significant, *χ*^2^(25) = 454.27, *p* < .001, as expected with large samples. Other fit indices indicated an excellent model fit: Comparative Fit Index (CFI) = .975, Tucker–Lewis Index (TLI) = .958, Root Mean Square Error of Approximation (RMSEA) = .055 [90% CI (.051,.060)], and Standardized Root Mean Square Residual (SRMR) = .050. These indices are consistent with recommended thresholds for good model fit (CFI and TLI >.95, RMSEA <.06, SRMR <.08 (17).

The latent variable “General Psychological Distress” was defined by the Edinburgh Postnatal Depression Scale (EPDS; standardized loading = .830, *p* < .001) and PROMIS Anxiety score (standardized loading = .957, *p* < .001). The “COVID-19-Specific Fear” latent variable was defined by three indicators: perceived threat to own life (standardized loading = .723, *p* < .001), perceived threat to unborn baby's life (.933, *p* < .001), and worry about harm to the unborn baby (.781, *p* < .001). All factor loadings were statistically significant, supporting the construct validity of the latent variables.

Both general psychological distress and COVID-19-specific fear during pregnancy were significantly associated with shorter gestational age at birth. Specifically, higher general psychological distress predicted lower gestational age (standardized *β* = –.048, *p* = .002), and higher COVID-19-specific fear also predicted lower gestational age (standardized *β* = –.058, *p* < .001), after controlling for maternal age, income, and education. Among the covariates, higher maternal education was associated with longer gestational age (*β* = .053, *p* < .001), while older maternal age was slightly associated with shorter gestational age (*β* = –.088, *p* < .001). Household income was not a significant predictor (*p* = .719).

For infant birth weight, COVID-19-specific fear was significantly associated with lower birth weight (standardized *β* = –.058, *p* < .001), whereas general psychological distress was not a significant predictor (standardized *β* = –.007, *p* = .632). None of the demographic covariates were significantly associated with birth weight (all ps > .05).

General psychological distress and COVID-19-specific fear were positively correlated (standardized covariance = .419, *p* < .001), indicating moderate overlap between these constructs.

## 4 Discussion

This study aimed to disentangle the independent effects of maternal general psychological distress (GPD) and COVID-19-specific fear (CSF) during pregnancy on gestational age at birth and infant birth weight. Our first hypothesis, predicting an independent association between higher GPD and adverse birth outcomes, was partially supported. GPD independently predicted shorter gestational age (standardized *β* = –.048), but it did not demonstrate an independent association with infant birth weight once CSF was accounted for in the model. In contrast, our second hypothesis, which proposed that higher CSF would also independently predict adverse birth outcomes, was fully supported. Elevated levels of COVID-19-specific fear were significantly and independently associated with both shorter gestational age at birth (standardized *β* = –.058) and lower infant birth weight (standardized *β* = –.058), even after controlling for GPD and established demographic covariates. These findings underscore the critical importance of distinguishing GPD from context-specific fears. Although GPD and CSF were moderately correlated (standardized covariance = .419), their differential predictive capacity—particularly the unique and robust link between CSF and lower infant birth weight where GPD showed no independent effect—highlights CSF as a distinct and potent risk factor for adverse perinatal outcomes. Regarding covariates, higher maternal education was associated with longer gestational age and older maternal age with slightly shorter gestational age; other demographic covariates did not significantly predict the primary birth outcomes in the final model.

It is also important to consider the moderate positive correlation (standardized covariance = .419) observed between GPD and CSF. This shared variance suggests a complex interplay between these two constructs. On one hand, individuals with higher baseline levels of GPD may possess a cognitive or emotional vulnerability that predisposes them to developing more intense specific fears when faced with a novel, large-scale threat like the pandemic. On the other hand, the chronic, acute stress of pandemic-specific fears could erode psychological resources, thereby exacerbating or even triggering symptoms of general depression and anxiety. It is most likely that this relationship is transactional and bidirectional. While our model successfully isolates the unique predictive utility of each construct, future longitudinal studies are needed to elucidate the temporal dynamics of this relationship—that is, to understand how general distress and specific fears may mutually influence each other over the course of a prolonged crisis.

### 4.1 Theoretical implications and novel contributions

The findings of this study offer several important theoretical implications and novel contributions to our understanding of perinatal mental health and its impact on birth outcomes, particularly within the context of large-scale societal stressors.

First, this research provides robust empirical validation for the theoretically posited distinction between general psychological distress (GPD) and event-specific fear (CSF). While the conceptual difference between diffuse anxiety/depression and acute, threat-specific fear is well-established in general psychopathology, its empirical demonstration and relevance to tangible health outcomes in the perinatal period, especially during a crisis like the COVID-19 pandemic, has been less clear (18). The current study's ability, through structural equation modeling, to demonstrate that CSF exerts unique predictive power on adverse birth outcomes, even when GPD is accounted for, strongly supports the argument that these are not merely different severities of a single distress continuum but rather distinct, albeit related, psychological constructs with potentially different etiological significance.

Second, and perhaps most critically, the differential associations with birth outcomes, particularly the finding that CSF independently predicted lower infant birth weight while GPD did not (once CSF was controlled), suggest that GPD and CSF may operate through partially divergent psychobiological pathways. The established link between GPD (encompassing chronic anxiety and depression) and shorter gestational age may be mediated by pathways involving sustained dysregulation of the hypothalamic-pituitary-adrenal (HPA) axis, leading to altered cortisol profiles, and chronic low-grade inflammatory processes (19). These processes are thought to contribute to the premature initiation of the parturition cascade. The novel finding that CSF independently predicted both shorter gestational age and, uniquely, lower infant birth weight, invites deeper theoretical exploration. We hypothesize that these distinct outcomes may reflect divergent physiological responses. Fear, as an acute response to a perceived immediate and specific threat, is classically associated with pronounced activation of the sympathetic-adrenal-medullary (SAM) axis, resulting in surges of catecholamines (20). These findings are consistent with the theoretical possibility that such acute physiological responses may impact fetal growth (leading to lower birth weight) through mechanisms like transient but significant reductions in uteroplacental perfusion due to vasoconstriction (21). This SAM-driven pathway, potentially triggered by the intense and specific worries inherent in CSF (e.g., viral threat to the fetus, healthcare collapse), could operate distinctly from, or synergistically with, the more chronic HPA/inflammatory pathways associated with GPD. The impact of CSF on gestational age might reflect an overlap with GPD mechanisms or an additional pathway where intense, acute stress can also trigger premature labor.

Third, these findings necessitate a refinement of existing biopsychosocial models of prenatal stress and adverse birth outcomes. Current models often operationalize “stress” broadly. This study suggests that the nature and cognitive content of the stressor significantly matter. The specific, existentially threatening cognitions central to CSF—focused directly on the survival and well-being of the mother and infant within a high-threat environment—may engage distinct neurobiological and physiological cascades compared to the more diffuse, future-oriented worries characteristic of generalized anxiety, or the anhedonia and hopelessness of depression. This aligns with theories differentiating fear, as a response to clear and present danger, from anxiety, as apprehension about uncertain future threats (22).

Fourth, the results challenge a simplistic dose-response view where any form of psychological distress contributes additively to a general “allostatic load.” While CSF undoubtedly contributes to maternal allostatic load, its specific impact, particularly on birth weight, suggests an additional dimension of risk related to the acute, threat-imminent nature of the stressor. It highlights that the type of psychological experience, not just its overall intensity or chronicity, can have specific imprints on different facets of fetal development and the timing of birth. The robust and independent link between CSF and birth weight, an outcome often reflecting sustained intrauterine growth conditions, suggests that pandemic-specific fears may induce physiological states particularly disruptive to optimal fetal growth, beyond the effects captured by general distress measures.

Furthermore, the link between CSF and adverse outcomes may be mediated not only by psychobiological pathways but also by behavioral changes. Intense fear of viral exposure, for instance, could have led some individuals to delay or avoid necessary prenatal appointments, impacting the timely detection of complications. Similarly, pandemic-related stress and fear are linked to changes in nutrition, sleep patterns, and physical activity, all of which are known to influence fetal growth and gestational timing. Therefore, CSF may exert its influence through a dual mechanism: a direct physiological one via SAM axis activation and an indirect behavioral one through the adoption of maladaptive health behaviors driven by fear and anxiety. Future research should aim to measure these behavioral mediators to create a more comprehensive model of risk.

### 4.2 Implications, strengths, and methodological considerations

The distinct impact of CSF on adverse birth outcomes, independent of general psychological distress (GPD), carries significant clinical and public health implications. Current perinatal mental health screening often prioritizes general depression and anxiety. Our findings strongly advocate for the supplementary assessment of event-specific fears, particularly during large-scale crises, as standard instruments may not fully capture this unique and potent source of risk. Consequently, intervention strategies should be tailored: while GPD may respond to established psychotherapies (such as CBT or mindfulness), CSF could benefit more from targeted psychoeducation and specific risk communication strategies. For example, rather than solely employing broad anxiety-reduction techniques, clinicians might prioritize providing concrete, data-driven information regarding hospital safety protocols to counteract fears of nosocomial infection, or offer specific reassurance about vertical transmission risks. For public health preparedness, these results underscore the need for proactive messaging and maternal care systems designed to address the specific anxieties of pregnant individuals during future crises, ensuring continuity of care and accessible psychosocial support.

The strengths of this study include the use of data from a large, prospective national cohort (the PdP study), which allowed for the examination of exposures before outcomes, thereby reducing recall bias. The application of structural equation modeling (SEM) (23) facilitated a nuanced analysis of the complex interrelationships between latent psychological constructs (GPD and CSF, defined by validated measures like the EPDS and PROMIS Anxiety scale) and birth outcomes, while statistically controlling for covariates. This approach provided a more sophisticated understanding than traditional regression methods by explicitly modeling the distinction and covariance between GPD and CSF. The clear conceptual and empirical differentiation between these two forms of distress is a core strength, supported by the good fit of our statistical model to the data. Methodologically, while the use of validated self-report measures is standard, future research could benefit from multi-method assessments. Addressing missing data with techniques beyond listwise deletion could also enhance the robustness of findings in subsequent investigations.

### 4.3 Limitations

Several limitations should be acknowledged. First, while our three-item measure for COVID-19-specific fear (CSF) demonstrated good internal consistency and clear factor loadings, future research could benefit from employing more comprehensively validated scales for pandemic-specific perinatal fear to further solidify these distinctions. Second, the psychological data were collected at a single time point during pregnancy. This cross-sectional assessment of distress does not capture the dynamic nature of these experiences. It is well-established that the impact of prenatal stress can vary depending on the timing of exposure during gestation, with different trimesters representing sensitive periods for different fetal developmental processes (24). The trajectory or chronicity of GPD and CSF across pregnancy may have differential or cumulative effects that our model could not assess. Future longitudinal research is needed to track these psychological states across all trimesters to disentangle the specific impact of stressor timing on birth outcomes. Third, while we proposed distinct psychobiological pathways, this study did not include direct biological measures (e.g., cortisol, inflammatory markers), thus the mechanistic links remain inferential. Finally, our handling of missing data via listwise deletion resulted in a substantial reduction in sample size and introduced a potential for selection bias, as noted in our comparison between the complete-case and incomplete-case samples. The final analytic sample was slightly older and had a higher proportion of English-speaking participants. Consequently, the exclusion of younger or less educated participants means our results might not fully capture the experiences of the most vulnerable populations, who often face unique financial or systemic stressors. Despite controlling for several covariates, the possibility of unmeasured confounders, such as the direct impact of maternal COVID-19 infection severity or specific nuances of healthcare disruptions experienced by individuals, cannot be entirely excluded (25).

## 5 Conclusion

This study provides evidence that maternal CSF during pregnancy represents a distinct and potent risk factor for adverse birth outcomes, specifically shorter gestational age and lower infant birth weight, independent of GPD. While GPD also predicted shorter gestational age, its effect on birth weight was not significant when CSF was accounted for. These findings underscore the critical importance of recognizing and assessing event-specific fears alongside broader measures of anxiety and depression in pregnant individuals. Recognizing ‘Event-Specific Fear’ as a distinct psychological construct is critical not only for the current context but for any future public health emergency (e.g., natural disasters, future pandemics).The unique predictive power of CSF suggests distinct underlying psychobiological pathways that warrant further investigation. Ultimately, a nuanced understanding of how different facets of psychological distress impact perinatal health is crucial for developing targeted screening protocols, tailored interventions, and effective public health strategies to mitigate the adverse consequences of pandemic-related stress, and future large-scale stressors, on both maternal and infant well-being.

## Data Availability

Publicly available datasets were analyzed in this study. This data can be found here: https://osf.io/ha5dp/.
